# Sex-differential effect of waist circumference on new-onset cerebral infarction: a nationwide cohort study

**DOI:** 10.3389/fneur.2024.1448428

**Published:** 2024-10-09

**Authors:** Sang Min Lee, Minha Hong, Jae-Hong Ryoo

**Affiliations:** ^1^Department of Psychiatry, Kyung Hee University Hospital, Kyung Hee University College of Medicine, Seoul, Republic of Korea; ^2^Department of Psychiatry, Myongji Hospital, Hanyang University College of Medicine, Goyang, Republic of Korea; ^3^Department of Occupational and Environmental Medicine, Kyung Hee University Hospital, Kyung Hee University College of Medicine, Seoul, Republic of Korea

**Keywords:** waist circumference, cerebral infarction, cohort study, stroke, gender difference

## Abstract

**Introduction:**

Excessive abdominal adiposity represents a cardiovascular disease risk factor. Waist circumference (WC) reflects abdominal adiposity and is known as an easy-to-measure indicator of obesity. This study aimed to evaluate the relationship between WC level and the development of cerebral infarction in Koreans.

**Methods:**

209,442 Koreans were included among the general population registered in the National Health Information Database. Depending on the degree of WC, the possibility of cerebral infarction was tracked for 4.37 person-years. Identification of patients with cerebral infarction was confirmed through the diagnostic code ICD I63 of inpatient or outpatient. Participants’ data were analyzed by sex. The hazard ratios (HRs) and confidence interval (CI) for cerebral infarction were calculated using the Cox proportional hazards model.

**Results and discussion:**

Between 2009 and 2013, 2,403 cases (1.15%) of cerebral infarction occurred during the follow-up period of 915,223.6 person-years. The HRs (95% CI) for incident cerebral infarction in men was adjusted for multiple covariates, and comparison of WC levels second, third, and fourth quartile with the first quartile showed 1.10 (0.94–1.28), 1.11 (0.95–1.30), and 1.24 (1.07–1.45), respectively (P for trend 0.045). This association was not significant in women (P for trend 0.619). The severity of WC levels in men is significantly associated with the risk of developing cerebral infarction in Koreans. This finding indicates that other measurements for excessive adipose visceral tissue, except abdominal circumference, need to be taken into account to identify the risk of cerebral infarction in women.

## Introduction

Obesity is associated with high blood pressure, dyslipidemia, and diabetes, all of which increase the risk of cardiovascular disease (CVD). Similar to other countries, this phenomenon is also on the rise in the Republic of Korea (1–3). Body mass index (BMI) is the most widespread and standardized tool among body fat-induced health risk assessment tools; however, it has limitations in the inability to distinguish body fat mass from lean body mass and reflect body fat distribution (4). An increased risk for CVD is known to be highly associated with abdominal or visceral fat. Previous studies have shown that body fat is more correlated with waist circumference (WC) (5, 6). WC is a simple-to-measure anthropometric index that reflects obesity, and abdominal fat (7, 8).

It has been shown that metabolic syndrome is closely linked to the occurrence and recurrence of stroke (9). People with metabolic syndrome have an increased risk of ischemic stroke even if the risk factors are adjusted (10, 11). WC is the most powerful tool for predicting metabolic syndrome, and while studies on the association between WC and ischemic stroke are available, they are limited (12, 13).

Thus, this study aimed to confirm the relationship between WC and clinically diagnosed cerebral infarction through a data analysis of a large retrospective cohort of South Koreans.

## Materials and methods

### Data sources

The South Korean government operates the National Health Insurance system covering approximately 97% of the entire population (14). Additionally, the majority of South Koreans aged 40 years and above take a mandatory medical health checkup at least every 2 years. All the data from medical health checkups were collected and stored by the National Health Insurance Corporation. A sampled database with personal identifiers removed is made available for research through the National Health Insurance Service (NHIS). The NHIS database sampling randomly selected 2.2% of the total South Korean population in 2002, and a systematic stratified random sampling was performed using the total annual medical expenses (14). Data on health checkups from the National Health Insurance Corporation (NHIC) were coupled with information on the development of cerebral infarction from Statistics Korea.

The study protocol was approved, and informed consent was waived by the institutional review board of Kyung Hee University Hospital (KHUH 2018–12-020). This study followed the Strengthening the Reporting of Observational Studies in Epidemiology (STROBE) reporting guidelines (see Supplementary materials) and was performed in accordance with other relevant guidelines and regulations.

### Study participants

**Figure 1:**
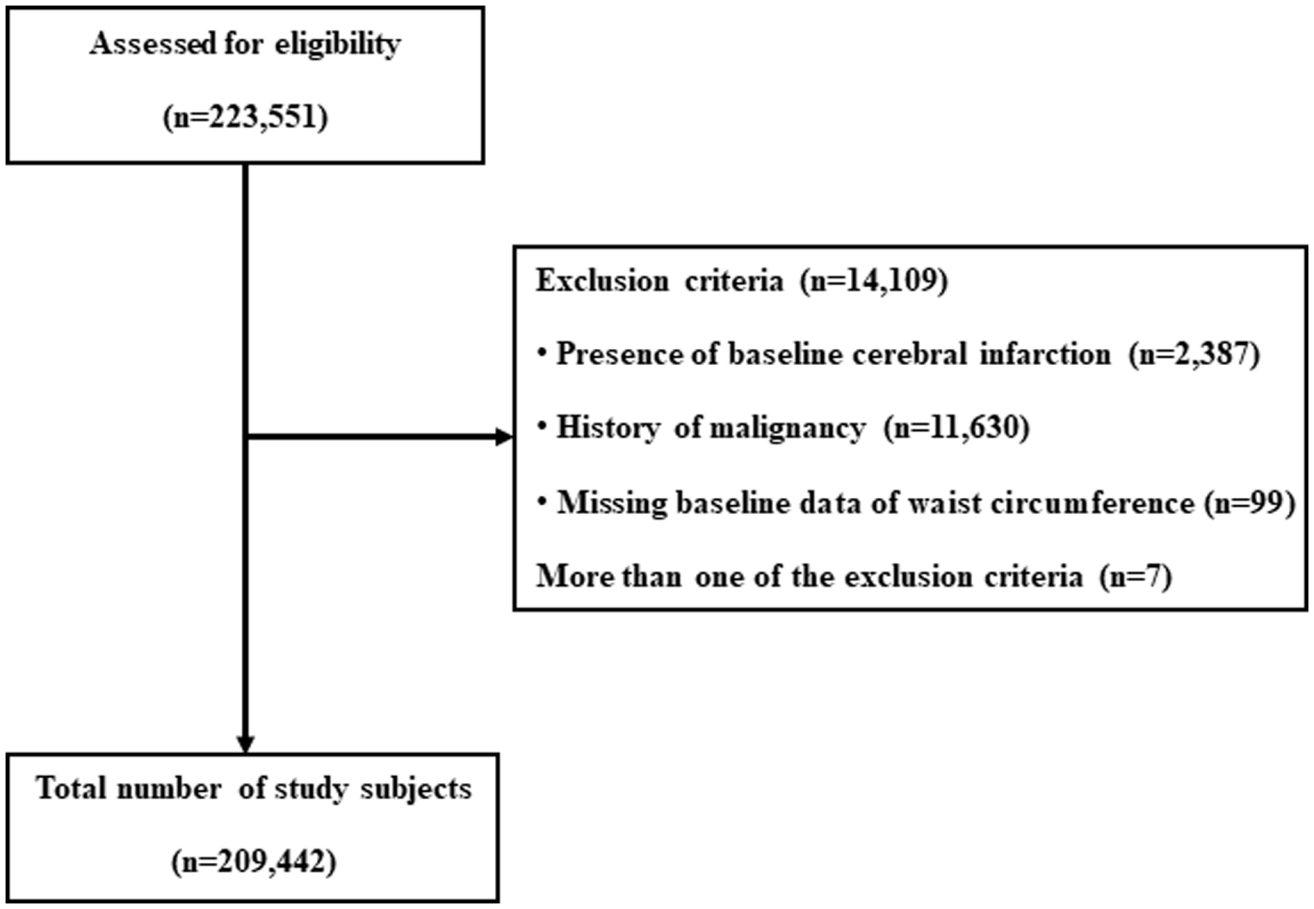
Flowchart of enrolled study subjects.

In 2009, a total of 223,551 medical health checkup records were analyzed. Data for 2,387 individuals with a history of cerebral infarction (ICD-I63) between 2002 and the date of the medical health examination in 2009 were excluded. Additional exclusion criteria included: missing information on baseline WC in 2009 (*n* = 99), a cancer diagnosis (ICD C00-C97) between 2002 and the medical health examination date (*n* = 11,630), and individuals meeting more than one of the abovementioned criteria (*n* = 7). Cancer patients are typically considered to have a severe disease with a potentially short life expectancy. In addition, considering the characteristics of the cancer itself and the risk of cerebral infarction that may occur during the cancer treatment process, we excluded factors that may affect the dependent variable from the cohort. Data from 209,442 individuals were finally included in the analysis and observed for the development of cerebral infarction. The total follow-up period was 915,223.6 person-years, and the average follow-up period was 4.37 (standard deviation [SD], 0.48) person-years (Figure 1).

### Health survey examinations and laboratory measurements

National health checkups are mandatory in the Republic of Korea, and all employees undergo health checkups every 1–2 years according to the Korean Occupational Safety and Health Act. The health checkup by the NHIC has two stages. The first stage involves a screening to identify disease among the non-symptomatic general population. The second stage includes a more detailed examination with a questionnaire on lifestyle or past medical history, physical activity, anthropometric measurements, smoking amount (pack-years) calculated from the smoking-related questionnaire, and alcohol intake (at least >3 times/week). Physical activity is defined as at least >30 min/day for >4 days/week, or vigorous-intensity activity (at least >20 min/day for >4 days /week). Additional measurements include BMI (weight (Kg)/height^2^ (m^2^)), blood pressures (both systolic and diastolic), and laboratory tests, which encompass fasting blood glucose, total cholesterol, triglyceride, high-density lipoprotein (HDL) cholesterol, low-density lipoprotein (LDL) cholesterol, serum creatinine (SCr), aspartate aminotransferase (AST), alanine aminotransferase (ALT), and *γ*-glutamyltransferase (GGT). The estimated glomerular filtration rate (eGFR) was measured using the Chronic Kidney Disease Epidemiology Collaboration (CKD-EPI) equation (15).

### Outcome definitions

The National Health Insurance database and diagnosis data from Statistics Korea were linked. In this study, the entry date was defined as the first health checkup since 2009, with the last follow-up date for the diagnosis of cerebral infarction set as 31 December 2013. The diagnosis of cerebral infarction was defined as in ICD-I63. In a previous study using health insurance claim data, the accuracy of the diagnosis of cerebral infarction (I63) using the ICD code was 83.4% (16). The development of cerebral infarction was the primary clinical endpoint of interest (17). In the Republic of Korea, cerebral infarction uses the I63 code diagnosed by a doctor. Cerebral infarction was considered to have occurred if at least one diagnostic code was present in either the hospitalization or outpatient records.

### Statistical analysis

Continuous variables were represented by means ± (standard deviation) or medians (interquartile range), and categorical variables were represented as percentages of the number.

The one-way ANOVA and chi-square-test were used to analyze the statistical differences between characteristics of the study participants at registration based on their WC quartile.

Person-years were calculated as the sum of follow-up time from baseline to the diagnosis of cerebral infarction or until 31 December 2013, with cases of death defined as censoring. Missing data were minimal, with a missing rate of less than 0.00001%, and these were excluded from statistical analysis.

Cox proportional hazards models were used to assess the associations between incident cerebral infarction and quartile groups based on WC levels. Adjusted hazard ratios (HRs) and 95% confidence interval (CI) for incident cerebral infarction were obtained by comparing the upper three quartiles of WC with the lowest quartile. Models were adjusted for multiple confounding factors. Possible confounding variables between WC and incident cerebral infarction (such as age, systolic BP, fasting blood glucose, LDL-cholesterol, GGT, eGFR, smoking amount (pack-years), alcohol intake, and physical activity) were included in the multivariable models. The research methodology of a previous study on metabolic syndrome using the same data source was referred to for analysis (18). The proportional hazard assumption was evaluated to test the validity of the Cox proportional hazards models. The proportional hazard assumption was assessed using log-minus-log survival function and found to be graphically unviolated. *p-*values <0.05 were considered to be statistically significant. All statistical analyses were performed using SAS (version 9.4, SAS Institute, Cary, NC, United States).

## Results

During a total follow-up of 915,223.6 person-years, there were 2,403 incident cases of cerebral infarctions (1.15%) reported from 2009 to 2013.

Tables 1, 2 outline the baseline characteristics of the participants according to the quartile groups of men’s and women’s baseline WC levels, respectively. There were statistically significant differences between all of the variables except SCr and quartile groups of men’s baseline WC levels and between all the variables except SCr and alcohol intake and quartile groups of women’s baseline WC levels. There was no significant difference in SCr levels between men and women, nor was there a significant difference in alcohol intake among women.

**Table 1:** Baseline characteristics of participants according to quartile groups of waist circumference levels in men (*N* = 118,356).

Characteristics	Waist circumference				
	Quartile 1 (<80, *n* = 27,599)	Quartile 2 (≥80, <85, *n* = 32,476)	Quartile 3 (≥85, <90, *n* = 30,306)	Quartile 4 (≥90, *n* = 27,975)	*P*-for trend*
Person-year (total)	119,411.2	141,082.8	131,561.0	121,242.4	
Person-year (average)	4.33 ± (0.54)	4.34 ± (0.48)	4.34 ± (0.48)	4.33 ± (0.53)	
Age (years)	57.1 ± (8.8)	56.4 ± (8.1)	56.9 ± (8.1)	57.9 ± (8.5)	<0.001
BMI (kg/m^2^)	21.2 ± (2.0)	23.3 ± (1.7)	24.8 ± (1.7)	26.9 ± (2.3)	<0.001
Systolic BP (mmHg)	122.8 ± (14.9)	125.7 ± (14.4)	127.3 ± (14.2)	129.7 ± (14.5)	<0.001
Diastolic BP (mmHg)	76.7 ± (9.7)	78.5 ± (9.6)	79.5 ± (9.7)	80.8 ± (9.9)	<0.001
Total cholesterol (mg/dL)	190.3 ± (34.7)	196.6 ± (36.2)	198.4 ± (36.6)	199.3 ± (37.5)	<0.001
Triglyceride (mg/dL)	98 (70–139)	123 (87–178)	138 (97–198)	152 (108–218)	<0.001
HDL-cholesterol (mg/dL)	57.6 ± (32.3)	53.4 ± (27.7)	51.6 ± (28.6)	50.4 ± (30.0)	<0.001
LDL-cholesterol (mg/dL)	111.3 ± (38.7)	115.3 ± (38.6)	115.5 ± (37.8)	114.6 ± (39.2)	<0.001
Fasting blood glucose (mg/dL)	98.8 ± (24.1)	102.1 ± (26.9)	103.8 ± (26.9)	107.5 ± (29.8)	<0.001
SCr (mg/dL)	1.26 ± (1.57)	1.40 ± (1.92)	1.38 ± (1.88)	1.27 ± (1.62)	0.598
eGFR (mL/min per 1.73m^2^)	82.4 ± (20.3)	80.2 ± (21.8)	79.5 ± (21.5)	79.0 ± (20.1)	<0.001
AST (U/L)	24 (20–29)	24 (20–29)	25 (21–31)	26 (22–33)	<0.001
ALT (U/L)	20 (15–26)	22 (17–30)	25 (19–34)	28 (21–39)	<0.001
GGT (U/L)	26 (18–40)	31 (21–51)	25 (24–58)	42 (28–68)	<0.001
Smoking amount (pack-year)	13.2 ± (15.3)	13.1 ± (15.2)	14.0 ± (16.1)	15.0 ± (17.5)	<0.001
Alcohol intake (%)	6,140 (22.5)	7,443 (23.2)	7,268 (24.3)	7,227 (26.1)	<0.001
Physical activity (%)	4,843 (17.9)	5,925 (18.7)	5,369 (18.1)	4,801 (17.5)	0.002
Development of cerebral infarction (%)	296 (1.07)	367 (1.13)	373 (1.23)	457 (1.63)	<0.001

Data are means (standard deviation), medians (interquartile range), or percentages. ^*^P-value by ANOVA test for continuous variables and chi-square test for categorical variables.

**Table 2:** Baseline characteristics of participants according to quartile groups of waist circumference levels in women (*N* = 91,086).

Characteristics	Waist circumference				
	Quartile 1 (<74, *n* = 23,480)	Quartile 2 (≥74, <79, *n* = 21,987)	Quartile 3 (≥79, <85, *n* = 24,224)	Quartile 4 (≥85, *n* = 21,395)	*P*-for trend*
Person-year (total)	103,216.4	96,939.5	107,099.8	94,670.5	
Person-year (average)	4.40 ± (0.40)	4.41 ± (0.41)	4.42 ± (0.44)	4.42 ± (0.52)	<0.001
Age (years)	55.7 ± (8.1)	57.7 ± (8.4)	59.7 ± (8.7)	62.0 ± (9.0)	<0.001
BMI (kg/m^2^)	21.3 ± (2.0)	23.1 ± (1.9)	24.6 ± (2.0)	27.1 ± (2.8)	<0.001
Systolic BP (mmHg)	118.8 ± (14.8)	122.5 ± (15.2)	125.3 ± (15.3)	128.9 ± (15.5)	<0.001
Diastolic BP (mmHg)	73.8 ± (9.6)	75.5 ± (9.8)	76.9 ± (9.8)	78.7 ± (9.9)	<0.001
Total cholesterol (mg/dL)	201.0 ± (35.8)	205.2 ± (37.6)	207.8 ± (38.5)	209.3 ± (39.4)	<0.001
Triglyceride (mg/dL)	89 (65–124)	104 (76–146)	117 (85–164)	130 (94–181)	<0.001
HDL-cholesterol (mg/dL)	62.1 ± (36.2)	58.8 ± (35.8)	56.8 ± (34.8)	55.3 ± (32.5)	<0.001
LDL-cholesterol (mg/dL)	120.6 ± (37.8)	124.6 ± (39.8)	125.9 ± (39.5)	125.6 ± (38.1)	<0.001
Fasting blood glucose (mg/dL)	93.2 ± (17.1)	96.1 ± (19.9)	98.7 ± (22.3)	103.4 ± (26.9)	<0.001
SCr (mg/dL)	0.90 ± (0.95)	0.92 ± (1.02)	0.91 ± (0.94)	0.92 ± (0.99)	0.271
eGFR (mL/min per 1.73m^2^)	83.6 ± (18.8)	82.5 ± (19.0)	81.1 ± (18.6)	79.2 ± (19.2)	<0.001
AST (U/L)	22 (19–26)	22 (19–27)	23 (19–27)	23 (20–29)	<0.001
ALT (U/L)	17 (13–21)	18 (14–24)	19 (15–26)	21 (16–29)	<0.001
GGT (U/L)	16 (12–21)	17 (13–24)	19 (14–27)	21 (16–31)	<0.001
Smoking amount (pack-year)	0.22 ± (2.02)	0.22 ± (2.26)	0.24 ± (2.49)	0.27 ± (2.41)	0.025
Alcohol intake (%)	533 (2.3)	549 (2.6)	572 (2.4)	487 (2.3)	0.850
Physical activity (%)	3,557 (15.5)	3,504 (16.3)	3,694 (15.5)	2,782 (13.3)	<0.001
Development of cerebral infarction (%)	123 (0.52)	181 (0.82)	265 (1.09)	341 (1.59)	<0.001

Data are means (standard deviation), medians (interquartile range), or percentages. ^*^P-value by ANOVA test for continuous variables and chi-square test for categorical variables. ^*^P-value by ANOVA test for continuous variables and chi-square test for categorical variables.

The participants with incident cerebral infractions were older (67.7 vs. 57.7 years) and more likely to have a less favorable metabolic profile at baseline than those who did not develop a cerebral infarction. As anticipated, both groups showed statistically significant differences in all clinical variables except for BMI, total cholesterol, LDL-cholesterol, SCr, and AST (see Supplementary Table S1).

HRs and 95% CI of cerebral infarction occurrence according to the baseline WC levels in men and women are identified in Tables 3, 4, respectively. In the unadjusted model, the HRs and 95% CI for incident cerebral infarction in men comparing quartiles 2, 3, and 4 vs. quartile 1 (reference group) were 1.05 (0.00–1.22), 1.14 (0.98–1.33), and 1.52 (1.31–1.76), respectively (P for trend <0.001). For women, the HRs and 95% CI for incident cerebral infarction comparing the upper quartiles of WC vs. quartile 1 (reference group) were 1.56 (1.24–1.96), 2.06 (1.66–2.55), and 2.99 (2.43–3.67), respectively (P for trend <0.001).

**Table 3:** Hazard ratios (HRs) and 95% confidence intervals (CI) for the incidence of cerebral infarction according to the quartile groups of waist circumference levels in men.

	Person-year	Incidence cases	Incidence density (per 10,000 person-year)	HR (95% CI)	
				Unadjusted	Multivariable adjusted model*
Waist circumference					
Quartile 1	119,411.2	296	24.8	1.00 (reference)	1.00 (reference)
Quartile 2	141,082.8	367	26.0	1.05 (0.90–1.22)	1.11 (0.94–1.29)
Quartile 3	131,561.0	373	28.4	1.14 (0.98–1.33)	1.15 (0.95–1.31)
Quartile 4	121,242.4	457	37.7	1.52 (1.31–1.76)	1.25 (1.07–1.46)
*P* for trend				<0.001	0.035
Age					1.09 (1.08–1.10)
Systolic BP					1.010 (1.007–1.014)
Fasting blood glucose					1.005 (1.004–1.007)
LDL-cholesterol					1.002 (1.000–1.003)
GGT					1.001 (1.000–1.002)
eGFR					0.996 (0.994–0.999)
Smoking amount					1.007 (1.005–1.010)
Alcohol intake					1.000 (0.883–1.132)
Physical activity					0.881 (0.769–1.010)

^*^Multivariable adjusted model was adjusted for age, systolic BP, fasting blood glucose, LDL-cholesterol, GGT, eGFR, smoking amount (pack-year), alcohol intake, and physical activity.

**Table 4:** Hazard ratios (HRs) and 95% confidence intervals (CI) for the incidence of cerebral infarction according to the quartile groups of waist circumference levels in women.

	Person-year	Incidence cases	Incidence density (per 10,000 person-year)	HR (95% CI)	
				Unadjusted	Multivariable adjusted model*
Waist circumference					
Quartile 1	103,216.4	123	11.9	1.00 (reference)	1.00 (reference)
Quartile 2	96,939.5	181	18.7	1.56 (1.24–1.96)	1.21 (0.96–1.53)
Quartile 3	107,099.8	265	24.7	2.06 (1.66–2.55)	1.23 (0.99–1.53)
Quartile 4	94,670.5	341	36.0	2.99 (2.43–3.67)	1.28 (1.03–1.59)
*P* for trend				<0.001	0.584
Age					1.11 (1.10–1.12)
Systolic BP					1.016 (1.012–1.019)
Fasting blood glucose					1.007 (1.006–1.009)
LDL-cholesterol					1.002 (1.000–1.003)
GGT					1.001 (0.998–1.003)
eGFR					0.998 (0.994–1.002)
Smoking amount					1.001 (0.983–1.003)
Alcohol intake					0.696 (0.383–1.265)
Physical activity					0.817 (0.657–1.016)

^*^Multivariable-adjusted model was adjusted for age, systolic BP, fasting blood glucose, LDL-cholesterol, GGT, eGFR, smoking amount (pack-year), alcohol intake, and physical activity.

In the multivariable-adjusted model, associations remained statistically significant in men; however, for women, they were no longer significant. The adjusted HRs and 95% CI for incident cerebral infarction in men were 1.10 (0.94–1.28), 1.11 (0.95–1.30), and 1.24 (1.07–1.45), respectively (P for trend 0.045) (Table 3). Among women, the adjusted HRs and 95% CI were 1.22 (0.96–1.54), 1.23 (0.99–1.53), and 1.28 (1.03–1.59), respectively (P for trend 0.619) (Table 4). The authors also examined the associations between men and women, finding that both the unadjusted and multivariable-adjusted models remained statistically significant (data not shown).

## Discussion

This study confirmed that the risk of cerebral infarction as quartiles of WC increased during the 5-year tracking period through a nationwide cohort among South Koreans. To the best of our knowledge, this is the first cohort study to reveal sex differences in the relationship between WC and a clinically diagnosed single-diagnosis code of cerebral infarction in the general population.

In two previous cohort studies conducted in the Republic of Korea, WC level was shown to be associated with stroke (19, 20). A cohort study found a positive correlation between WC with myocardial infarction and ischemic stroke, which predicted CVD events better than BMI (19). A second study revealed that WC and body weight were positively associated with heart attack, stroke, and all-cause mortality (20). However, compared to our study which shows statistically significant differences only in men, both these studies did not analyze men and women separately. In addition to the I63 ICD code used in this study for ischemic stroke, differences in the I64 ICD code are also included in other studies.

A prospective study in Finland showed that men in the highest quartile of WC or waist-to-hip ratio had a high risk of total and ischemic stroke, but not in women. The risk of hemorrhagic stroke in women showed a U-shaped relationship with BMI (21). In a cohort study including 5,474 Japanese individuals, the risk of CVD and stroke was higher among those with WC greater than 84 cm and less than 70, and this tendency was only seen in women. After adjustment for hypertension, diabetes, and hypercholesterolemia, the results were shown to be statistically not significant (22). A study of 54,717 people at MORGAM cohort found a higher association between abdominal fat measurements (WC, waist-to-hip ratio, and waist-to-height ratio) and stroke risk in men (23). Abdominal fat was more strongly associated with cerebral hemorrhage than ischemic cerebral infarction in a prospective cohort of Chinese women, although there was no difference in the predictive value for stroke (24).

Several recent studies indicate that there may be gender differences in the relationship between WC and the risk of stroke (23). In this regard, biological differences in obesity, metabolic syndrome, sex hormones, and cardiovascular disease between men and women are mainly discussed, and it is known that hormones, fat distribution, and physiological responses play an important role in these differences (25). Men tend to accumulate fat mainly in the abdomen, while women tend to accumulate more fat in the subcutaneous fat of the buttocks, thighs, etc., before menopause, and visceral fat tends to increase after menopause (26). Differences in muscle and fat distributions between men and women revealed that WC was the best predictor in older men, and the waist-to-hip ratio was the least predictive in women (27). A recent study suggests that WC, waist-to-height ratio, and conicity index are predictive in men in measuring excessive adipose visceral tissue; however, age, sagittal diameter, conicity index, and neck circumference are better predictors for women rather than WC (28). Compared to previous studies, this present study included a large population, in which the risk of WC and cerebral infarction was significant only in men and there was no difference in women after adjusting for other risk factors.

Differences in sex hormones between men and women are also involved, with estrogen playing a role in protecting women from vascular disease, which has the effect of reducing the risk of stroke before menopause. Testosterone can promote visceral fat accumulation and metabolic abnormalities in men, which may be associated with an increased risk of cerebral infarction (29). In addition, there are differences in the developmental mechanisms of metabolic syndrome between men and women, and it is known that men can develop metabolic abnormalities such as insulin resistance, hypertension, and hyperlipidemia more quickly when their waist circumference increases, which may lead to differences in waist circumference and the risk of cerebral infarction. Women have a relatively low risk of metabolic syndrome before menopause, but after menopause, they experience an increase in metabolic risk similar to that of men (30, 31). Additional research is needed to determine whether these biological differences between men and women contribute to differences in cardiovascular risk.

The study has several limitations. First, among the many methods for measuring abdominal fat, this study used only WC as this was the information available from the Korean national health checkup. The use of measures other than WC has been shown to be more effective in predicting women’s risk of cerebral infarction. Second, collection bias may exist because raw data were collected from medical examinations and related surveys in this study. Third, the follow-up period of the study subjects was only 4.37 years, limiting the understanding of the long-term impacts of WC on cerebral infarction. Further studies involving the extension of the follow-up period are needed. Fourth, although the statistical analysis of this study confirmed the significant results using quartile groups, there is clearly an aspect that must be considered for residual confounding because of the complexity that exists between WC and other cardiovascular risk factors and cerebral infarction. Fifth, this cohort has no data on the recurrence of cerebral infarction and therefore cannot distinguish between initial and recurrent strokes. Future research studies will require efforts to improve these aspects by linking them with hospital data.

## Conclusion

Waist circumference (WC), one of the abdominal fat markers, was graded and found to be significantly related to the risk of cerebral infarction, regardless of other vascular risk factors. WC is more predictable in men than in women. According to this study, the higher WC was regarded as a risk factor of cerebral infarction. Additionally, it was revealed that cerebral infarction showed increased risk as quartiles of WC level increased in men. From a public health point of view, WC can be utilized as a clinical and epidemiological tool for the early identification of high-risk individuals.

## Data Availability

The data analyzed in this study is subject to the following licenses/restrictions: The data that support the findings of this study are available from the National Health Insurance Service in South Korea but restrictions apply to the availability of these data, which were used under license for the current study, and so are not publicly available. Data are however available from the corresponding author upon reasonable request and with permission of the National Health Insurance Service in South Korea. Requests to access these datasets should be directed to https://nhiss.nhis.or.kr/bd/ay/bdaya001iv.do.
