# Supplementary material for: Sex-differential effect of waist circumference on new-onset cerebral infarction: a nationwide cohort study
Source: Front Neurol. 2024 Oct 9;15:1448428. doi: 10.3389/fneur.2024.1448428 (PMC11496052; doi:10.3389/fneur.2024.1448428)
Supplement: Supplementary file 1 [file Table_1.DOC]

**Table S1. Comparison between participants with and without incident cerebral infarction.**

| **Characteristic** | **Without incident cerebral infarction (N=207,039)** | **With incident cerebral infarction (N=2,403)** | ***P*-value^*^** |
| --- | --- | --- | --- |
| Age (years) | 57.7 ± (8.6) | 66.7 ± (9.4) | <0.001 |
| Sex |  |  | <0.001 |
| Male (%) | 116,863 (56.4) | 1,493(62.1) |  |
| Female (%) | 90,176 (43.6) | 910 (37.9) |  |
| BMI (kg/m^2^) | 24.0 ± (2.9) | 24.1 ± (2.9) | 0.196 |
| Systolic BP (mmHg) | 125.2 ± (15.1) | 131.9 ± (17.3) | <0.001 |
| Diastolic BP (mmHg) | 77.7 ± (9.9) | 80.1 ± (10.8) | <0.001 |
| Total cholesterol (mg/dL) | 200.4 ± (37.4) | 201.4 ± (40.0) | 0.229 |
| Triglyceride (mg/dL) | 142.0 ± (94.3) | 154.9 ± (97.8) | <0.001 |
| HDL-cholesterol (mg/dL) | 55.4 ± (32.3) | 52.6 ± (29.8) | <0.001 |
| LDL-cholesterol (mg/dL) | 118.5 ± (39.0) | 119.2 ± (39.9) | 0.407 |
| Fasting blood glucose (mg/dL) | 100.6 ± (25.1) | 108.7 ± (35.5) | <0.001 |
| SCr (mg/dL) | 1.15 ± (1.49) | 1.14 ± (1.28) | 0.600 |
| eGFR (mL/min per 1.73m^2^) | 80.9 ± (20.2) | 74.3 ± (19.3) | <0.001 |
| AST (U/L) | 26.5 ± (16.3) | 27.0 ± (24.4) | 0.413 |
| ALT (U/L) | 25.4 ± (19.2) | 24.4 ± (22.2) | 0.025 |
| GGT (U/L) | 39.1 ± (53.9) | 43.8 ± (60.7) | <0.001 |
| Smoking amount (pack-year) | 7.8 ± (13.8) | 10.3 ± (16.6) | <0.001 |
| Alcohol intake (%) | 14.6 | 16.5 | 0.010 |
| Physical activity (%) | 16.8 | 15.1 | 0.029 |

Data are expressed as means (standard deviation) or percentages.

^*^*P*-value by t-test for continuous variables and Chi square test for categorical variables.
